# Altered Gas6/sMerTK Balance in Gingival Crevicular Fluid Across Periodontal Health, Gingivitis, and Periodontitis: A Cross-Sectional Study

**DOI:** 10.3390/jcm15135044

**Published:** 2026-06-28

**Authors:** Nuray Ercan, Bilge Meracı Yıldıran, Zeynep Akgül, Esra Ateş Yıldırım, Özgür Mehmet Yis

**Affiliations:** 1 Department of Periodontology, Faculty of Dentistry, Bolu Abant Izzet Baysal University, 14030 Bolu, Turkey; bilgemeraci@ibu.edu.tr (B.M.Y.); zeynepakgul@ibu.edu.tr (Z.A.); esraates@ibu.edu.tr (E.A.Y.); 2 Department of Medical Biochemistry, Faculty of Medicine, Bolu Abant Izzet Baysal University, 14030 Bolu, Turkey; ozgurmehmetyis@ibu.edu.tr

**Keywords:** gingival crevicular fluid, Gas6, MerTK, efferocytosis, TAM receptors, periodontitis, resolution of inflammation

## Abstract

**Background/Objectives**: Periodontitis reflects a dysregulated inflammatory response in which impaired resolution and efferocytosis may play a central role. The TAM receptor MerTK and its ligand growth arrest-specific-6 (Gas6) are key regulators of these processes; however, their profile in gingival crevicular fluid (GCF) across the periodontal disease spectrum remains unclear. This cross-sectional study aimed to evaluate GCF levels of Gas6 and soluble MerTK (sMerTK) in individuals with periodontal health, gingivitis, and periodontitis, and to examine their associations with clinical periodontal parameters. **Methods**: Eighty-one systemically healthy adults were enrolled into three groups: periodontal health, gingivitis, and periodontitis. Full-mouth clinical periodontal measurements were recorded. Gas6 and sMerTK levels were quantified by enzyme-linked immunosorbent assay. **Results**: sMerTK progressively decreased from periodontal health to gingivitis and periodontitis, with significant differences among all groups (*p* < 0.001). Gas6 total amount was higher in the diseased groups, whereas its concentration decreased. The Gas6/sMerTK ratio increased stepwise with disease severity (*p* < 0.001), showing positive correlations with clinical attachment loss and other clinical periodontal parameters, while sMerTK showed inverse correlations. **Conclusions**: These findings suggest that the local GCF Gas6/sMerTK balance is altered across periodontal states, primarily due to a marked reduction in sMerTK. The Gas6/sMerTK ratio may represent a potential exploratory indicator of periodontal inflammatory status, although longitudinal validation is required.

## 1. Introduction

Periodontitis is a biofilm-initiated chronic inflammatory disease in which destruction of the tooth-supporting connective tissue and alveolar bone is driven less by microbial burden alone than by a dysregulated and inadequately resolved host immune-inflammatory response [1,2]. Within this framework, the transition from gingivitis to periodontitis in susceptible individuals is increasingly understood not as a simple quantitative escalation of inflammation but as a qualitative failure of its resolution [1]. This view has directed attention toward the molecular mechanisms of inflammation resolution, in which efferocytosis—the immunologically silent clearance of apoptotic cells by macrophages—is central [3,4], as it prevents the accumulation of inflammatory cell debris and sustains a pro-resolving environment in inflamed tissues, including the periodontium [5].

Efferocytosis is governed in part by the TAM family of receptor tyrosine kinases (TYRO3, AXL, MerTK) and their bridging ligand growth arrest-specific 6 (Gas6), which tethers phosphatidylserine on apoptotic cells to the receptor and thereby enables macrophage recognition and engulfment [6,7,8]. Of the three receptors, MerTK is the principal efferocytic receptor on macrophages, and Gas6–MerTK signaling not only mediates apoptotic-cell uptake but also reinforces anti-inflammatory, pro-resolving macrophage programs [7,8]. Impaired efferocytosis consequently favors secondary necrosis and the persistent inflammation that characterizes chronic inflammatory disease [3,5].

Under oxidative and inflammatory stress, membrane MerTK is proteolytically shed, releasing a soluble ectodomain (sMerTK) that simultaneously depletes the surface efferocytic receptor pool and can act as a decoy by sequestering Gas6 [9,10,11]. The functional significance of this decoy effect depends on the local concentration of sMerTK in the surrounding microenvironment. In human atherosclerotic plaques, sMerTK correlates with necrosis and defective resolution, yet recombinant soluble Mer inhibits efferocytosis only at concentrations far exceeding those found in plasma [11]. Elevated sMerTK is therefore most reliably read as a marker of accelerated MerTK shedding and reduced surface-receptor function, with a directly competitive effect plausible only within the high local concentrations of an inflamed lesion. Consistent with this, circulating soluble Mer is increased and tracks disease activity and nephritis in systemic lupus erythematosus [12]; in rheumatoid arthritis, the soluble TAM receptors are also altered, although it is soluble Tyro3, rather than soluble Mer, that correlates with disease activity and bone destruction [13].

Saliva has been widely investigated as a non-invasive source of periodontal biomarkers, with host-derived analytes such as matrix metalloproteinase-8 and pro-inflammatory cytokines (e.g., interleukin-1β and interleukin-6) proposed for the detection and monitoring of periodontitis [14,15]. Investigation of the TAM pathway in periodontal disease is recent. Mendes et al. reported that salivary AXL, TYRO3, MerTK, and Gas6 were elevated in periodontitis relative to health and gingivitis; however, sMerTK was the weakest discriminator, reaching significance only in severe (stage III/IV) disease and showing the lowest diagnostic accuracy of the four analytes, which the authors attributed in part to the diverse tissue origins of salivary soluble TAM proteins [16]. Whole saliva integrates secretions from major and minor glands, mucosal transudate, and oral microbiota, and is thus an anatomically nonspecific window onto a localized periodontal lesion. Gingival crevicular fluid (GCF), by contrast, is a serum-derived inflammatory exudate sampled directly from the gingival sulcus, whose composition predominantly reflects the underlying periodontal tissue [2], and should provide a more proximal readout for macrophage-derived shed products such as sMerTK.

To our knowledge, no clinical study has quantified Gas6 or sMerTK in human GCF across periodontal health, gingivitis, and periodontitis. The aim of this cross-sectional study is therefore to evaluate GCF levels of Gas6 and sMerTK in individuals with periodontal health, gingivitis, and periodontitis, and to examine their associations with clinical periodontal parameters. The null hypothesis was that GCF Gas6 and sMerTK levels do not differ among individuals with periodontal health, gingivitis, and periodontitis, and are not associated with clinical periodontal parameters. The alternative hypothesis was that, relative to periodontal health, the inflamed gingival microenvironment of gingivitis and especially of periodontitis is associated with altered GCF Gas6 and increased GCF sMerTK, consistent with locally enhanced shedding of the macrophage efferocytic receptor MerTK.

## 2. Materials and Methods

### 2.1. Study Design

This cross-sectional comparative study was conducted among individuals attending the Department of Periodontology, Faculty of Dentistry, Bolu Abant Izzet Baysal University. The study protocol was approved by the Non-Interventional Clinical Research Ethics Committee of Bolu Abant Izzet Baysal University (Approval no: 2026/133) and registered at ClinicalTrials.gov (NCT07535177). All procedures were performed in accordance with the ethical principles of the Declaration of Helsinki. Before enrollment, all participants received verbal and written information about the study, and written informed consent was obtained from each participant. The study was reported in accordance with the Strengthening the Reporting of Observational Studies in Epidemiology (STROBE) guidelines.

### 2.2. Participants

**Figure 1:**
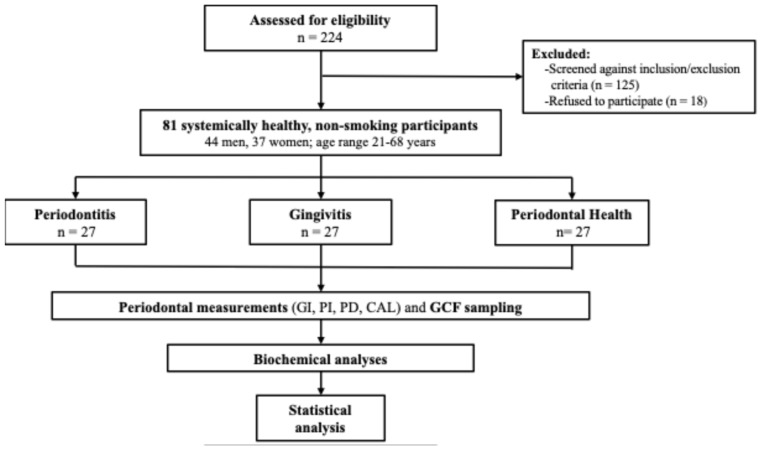
Flowchart of the study.

Systemically healthy, non-smoking individuals who consecutively presented for periodontal care between 25 March 2026 and May 2026 were screened for eligibility. A total of 81 participants were included in the study, with 27 individuals allocated to each group: periodontal health, gingivitis, and periodontitis. The study population consisted of 44 men and 37 women, aged 21–68 years (Figure 1).

The inclusion criteria were as follows:

- Age ≥ 18 years;
- Presence of at least 20 natural teeth, excluding third molars;
- Absence of systemic diseases that could affect periodontal tissues, including cardiovascular disease, atherosclerosis, immune-mediated disorders, rheumatoid arthritis, obesity, cancer, and diabetes mellitus;
- No antibiotic use within the preceding 3 months;
- No use of medications known to affect periodontal tissues;
- No infectious diseases such as HIV, HBV, or tuberculosis.

The exclusion criteria were as follows:

- Pregnancy or lactation;
- Periodontal treatment within the preceding 6 months;
- Current smoking.

### 2.3. Clinical Examination

All clinical measurements were performed by a single experienced examiner (N.E.) using a manual periodontal probe (Williams probe, Hu-Friedy, Chicago, IL, USA). Participants were assigned to one of three groups according to their periodontal status, based on the 2017 World Workshop classification of periodontal and peri-implant diseases and conditions [17,18].

The periodontal health group included individuals with probing depth (PD) ≤ 3 mm, bleeding on probing (BOP) < 10%, no interproximal clinical attachment loss (CAL) or radiographic bone loss (RBL), and clinically healthy gingiva on an intact periodontium [18]. The gingivitis group included individuals with PD ≤ 3 mm, BOP ≥ 30%, and no interproximal CAL or RBL [18]. The periodontitis group consisted of patients diagnosed with Stage III, Grade B periodontitis, characterized by interproximal CAL ≥ 5 mm, PD ≥ 6 mm, and tooth loss attributable to periodontitis of ≤ 4 teeth [17].

In the periodontitis group, attachment loss attributable to non-periodontal causes, including endodontic lesions draining through the marginal periodontium, cervical caries, traumatic gingival recession, or distal bone loss of second molars associated with third-molar extraction, was not interpreted as periodontitis.

The following clinical periodontal parameters were recorded: gingival index (GI), plaque index (PI), PD, and CAL. GI [19] and PI [20] were assessed at four surfaces per tooth (buccal, lingual/palatal, mesial, and distal). PD and CAL were measured at six sites per tooth: mesiobuccal, mid-buccal, distobuccal, mesiopalatal/lingual, mid-palatal/lingual, and distopalatal/lingual. CAL was calculated as the distance from the cemento-enamel junction to the base of the probeable pocket.

Radiographic bone loss was assessed on panoramic radiographs. BOP was used solely as a participant-level diagnostic criterion.

### 2.4. GCF Sampling

GCF samples were collected from interproximal sites (mesiobuccal or distobuccal) of two non-adjacent single-rooted teeth in each participant. Sampling sites were selected according to periodontal status: sites with GI < 1 and PD ≤ 3 mm in the periodontal health group, sites with GI ≥ 2 and PD ≤ 3 mm in the gingivitis group, and the deepest pockets with PD ≥ 5 mm and CAL ≥ 5 mm in the periodontitis group.

Before sampling, supragingival plaque was carefully removed, and the site was isolated with cotton rolls and gently air-dried. Standardized filter paper strips (PerioPaper, Oraflow Inc.) were inserted into the gingival sulcus until mild resistance was felt and left in place for 30 s. Strips contaminated with blood or saliva were discarded. The GCF volume of each strip was measured using a pre-calibrated electronic device (Periotron 8010, Oraflow Inc., Plainview, NY, USA). For each participant, the mean Periotron reading obtained from the two strips was calculated and then converted to GCF volume (µL) using the device calibration curve. The two strips obtained from each participant were pooled in the same Eppendorf tube and stored at −80 °C until biochemical analysis.

### 2.5. Biochemical Analysis

GCF levels of sMerTK and Gas6 were measured using commercial enzyme-linked immunosorbent assay (ELISA) kits according to the manufacturers’ instructions. All analyses were performed at the Department of Biochemistry Laboratory, Faculty of Medicine, Bolu Abant İzzet Baysal University.

For each participant, the pooled GCF sample was eluted from the paper strips by adding 500 µL of phosphate-buffered saline (PBS), followed by centrifugation at 5800 rpm for 5 min. The supernatant was collected and used for biochemical analysis. Gas6 was quantified using a Human Gas6 ELISA Kit (Novus Biologicals, LLC, Centennial, CO, USA; cat. no. KA2245; standard curve range: 125–8000 pg/mL), and sMerTK was quantified using a Human Mer DuoSet ELISA Kit (R&D Systems, Inc., Minneapolis, MN, USA; cat. no. DY6488; assay range: 156–10000 pg/mL). Optical density was measured at 450 nm using a microplate reader (Bio-Rad Spectrophotometer, Inc., Hercules, CA, USA), and biomarker concentrations were calculated from the corresponding standard curves. The intra- and inter-assay coefficients of variation were <10%. All biochemical analyses were performed by laboratory personnel who were blinded to the clinical group allocation of the samples; the samples were coded prior to analysis and decoded only after all measurements had been completed.

Biochemical data were expressed as both total amount per sampling period (sMerTK: pg/30 s; Gas6: ng/30 s) and concentration (sMerTK: pg/µL; Gas6: ng/µL). The total amount was defined a priori as the primary outcome, whereas concentration was considered a secondary, volume-adjusted measure.

### 2.6. Statistical Analysis

Sample size was calculated using G*Power software (version 3.1.9.6; Heinrich Heine University, Düsseldorf, Germany). The calculation was based on a one-way analysis of variance model with three groups (fixed-effects, omnibus), assuming a large effect size of f = 0.40, in accordance with Cohen’s conventions for analysis of variance [21], an α level of 0.05, and a statistical power of 85% (1 − β = 0.85). Under these assumptions, the minimum required total sample size was 75 participants (25 per group). To provide a margin above this minimum and to maintain balanced groups, 81 participants (27 per group) were enrolled.

All statistical analyses were performed using R software (version 4.5.0; R Foundation for Statistical Computing, Vienna, Austria). The normality of continuous variables was assessed using the Shapiro–Wilk test. Normally distributed quantitative variables were presented as mean ± standard deviation, whereas non-normally distributed variables were reported as median (minimum) and mean rank. Categorical variables were expressed as frequencies and percentages.

For comparisons among the three study groups, normally distributed quantitative variables were analyzed using one-way analysis of variance (ANOVA). Depending on variance homogeneity and the suitability of group comparisons, post-hoc analyses were performed using Tukey or Games–Howell tests. Non-normally distributed quantitative variables were compared using the Kruskal–Wallis H test, followed by Dunn’s test with Bonferroni correction for pairwise comparisons.

Associations between categorical variables and study groups were evaluated using Fisher’s exact test with Monte Carlo simulation. When required, multiple comparisons were performed using the Z test with Bonferroni correction. Correlations between non-normally distributed variables were assessed using Spearman correlation analysis, and correlation coefficients were reported with r values and corresponding significance levels.

Statistical significance was set at *p* < 0.05. Effect sizes were calculated using eta-squared (η^2^) or Cramér’s V, and 95% confidence intervals were obtained using bootstrap resampling and presented in the tables.

## 3. Results

### 3.1. Study Population

A total of 81 individuals were enrolled, equally distributed across the three groups (*n* = 27, per group). Sex distribution showed a statistically significant difference among the groups (*p* = 0.048). The periodontitis group had a higher proportion of males (74.1%) than females (25.9%), whereas the gingivitis and periodontally healthy groups both had a higher proportion of females (55.6%) than males (44.4%). Mean age differed significantly among the study groups (*p* < 0.001). Post-hoc comparisons showed that participants in the periodontitis group were significantly older than those in both the gingivitis and periodontally healthy groups, whereas age was comparable between the gingivitis and periodontally healthy groups (Table 1).

### 3.2. Clinical Periodontal Parameters

Clinical periodontal parameters showed significant differences among the study groups. GCF volume progressively increased from periodontally healthy individuals to those with gingivitis and periodontitis, with significant differences observed between all groups (*p* < 0.001). Similarly, PI and GI values were highest in the periodontitis group, followed by the gingivitis and periodontally healthy groups, and post-hoc comparisons revealed significant differences between all groups (*p* < 0.001). In contrast, PD and CAL values were significantly higher in the periodontitis group than in both the gingivitis and periodontally healthy groups, whereas no significant differences were observed between the gingivitis and periodontally healthy groups (*p* < 0.001) (Table 2).

### 3.3. Biochemical Parameters

Biochemical parameters showed significant differences among the study groups. The total amount of Gas6 was significantly higher in the periodontitis and gingivitis groups than in the periodontally healthy group, whereas no significant difference was observed between the periodontitis and gingivitis groups (*p* < 0.001). In contrast, Gas6 concentration was significantly lower in the periodontitis group than in both the gingivitis and periodontally healthy groups, while the gingivitis and periodontally healthy groups showed comparable values (*p* < 0.001). Both the total amount and concentration of sMerTK progressively decreased from periodontally healthy individuals to those with gingivitis and periodontitis, with significant differences observed between all groups (*p* < 0.001). Conversely, the Gas6/sMerTK ratio progressively increased across disease severity, with the highest values detected in the periodontitis group, followed by the gingivitis and periodontally healthy groups; post-hoc comparisons revealed significant differences between all groups (*p* < 0.001) (Table 3).

### 3.4. Correlation Analyses

Spearman correlation analysis revealed distinct associations between biochemical parameters and clinical periodontal measures. The Gas6/sMerTK ratio showed significant positive correlations with GCF volume and all clinical periodontal parameters, including PD, CAL, PI, and GI (r = 0.582–0.797, *p* < 0.001). In contrast, sMerTK total amount was negatively correlated with GCF volume and clinical periodontal parameters, with moderate-to-strong correlation coefficients (r = −0.609 to −0.789, *p* < 0.001). Gas6 total amount demonstrated weaker but significant positive correlations with PD, CAL, and PI, and moderate positive correlations with GCF volume and GI (r = 0.255–0.506, *p* ≤ 0.022). Among the biochemical parameters, the Gas6/sMerTK ratio was strongly and inversely correlated with sMerTK total amount (r = −0.933, *p* < 0.001) and positively correlated with Gas6 total amount (r = 0.641, *p* < 0.001). A moderate negative correlation was also observed between Gas6 and sMerTK total amounts (r = −0.453, *p* < 0.001). Exact coefficients and *p*-values are given in Table 4.

## 4. Discussion

To the best of our knowledge, this is the first study to evaluate Gas6 and sMerTK together in human GCF across periodontal health, gingivitis, and Stage III/Grade B periodontitis. Our findings indicate that the local Gas6/sMerTK balance changes markedly with disease severity. The Gas6/sMerTK ratio increased stepwise from periodontal health to gingivitis and periodontitis, mainly driven by a pronounced decrease in sMerTK rather than by a modest increase in Gas6. Since GCF volume is known to increase with inflammation [22], the total amount has been recommended as a less volume-dependent outcome measure in GCF biomarker studies [23]. In this context, the reduction in sMerTK in both total amount and concentration suggests that this decrease cannot be explained solely by dilution due to increased GCF volume. In contrast, Gas6 showed a divergent pattern, with a slight increase in total amount but a decrease in concentration, indicating that total amount may better reflect the local Gas6 response in inflamed periodontal sites.

The rationale for examining Gas6 and MerTK in periodontal disease rests on the role of efferocytosis in the resolution of inflammation, the failure of which is increasingly implicated in the gingivitis-to-periodontitis transition [1,3,4,5]. The Gas6–MerTK axis is a principal controller of this process: Gas6 bridges apoptotic cells to MerTK, the dominant macrophage efferocytic receptor, and the resulting signaling reinforces pro-resolving, anti-inflammatory programs while restraining NF-κB– and STAT1–driven output [6,7,8,24,25]. Their soluble forms are mechanistically informative because the functional effector is the membrane receptor: under inflammatory stress, MerTK undergoes ectodomain shedding (e.g., by ADAM17), which depletes the surface receptor pool and releases sMerTK—a fragment that can also sequester Gas6 as a decoy at high local concentrations—whereas soluble Gas6 reflects ligand availability within the exudate [9,10,11]. The local expression of TAM components in masticatory mucosa [26], the impairment of macrophage efferocytosis in periodontitis [5,27], and the protective Tyro3/Pros1 arm [28] support the plausibility of this axis operating within the periodontium. Because soluble analytes are surrogates of these membrane-level events, our cross-sectional measurements position Gas6 and sMerTK as biologically plausible participants in—and informative readouts of—the resolution axis, rather than as established causal drivers of disease.

The sMerTK finding was contrary to our initial hypothesis. Based on MerTK ectodomain shedding described in the atherosclerosis model, we had anticipated that the inflammatory periodontal microenvironment would increase sMerTK levels [11]. However, sMerTK levels were highest in periodontal health and lowest in periodontitis. This hypothesis was derived from atherosclerotic plaque and plasma data showing that soluble Mer increases with lesion progression and may represent an indicator, rather than a direct cause, of impaired MerTK-mediated efferocytosis [11]. The present findings suggest that this pattern observed in atherosclerotic lesions may not be directly extrapolated to the GCF microenvironment.

Although this finding may appear unexpected at first, it is not entirely inconsistent with the literature, as sMer does not exhibit a uniform biological pattern across inflammatory diseases. In systemic lupus erythematosus, circulating soluble Mer levels have been reported to increase and to reflect disease activity and nephritis [12]. In contrast, in rheumatoid arthritis, serum soluble Mer was not associated with disease activity or radiographic bone destruction, whereas soluble Tyro3 showed significant associations with these parameters [13]. The only previous evidence in the periodontal field is based on saliva samples; in that study, MerTK was higher in periodontitis but represented the weakest marker among the four TAM analytes and reached significance only in severe disease [16]. The authors attributed this finding to the potential contribution of multiple tissue sources to soluble TAM proteins in saliva [16]. Saliva integrates glandular, mucosal, and microbial inputs and is relatively distant from the periodontal lesion, whereas GCF is collected directly from the gingival sulcus/periodontal pocket microenvironment. Therefore, the more pronounced and opposite sMerTK gradient observed in GCF may reflect a local periodontal signal that is less clearly captured in saliva.

The decrease in GCF sMerTK levels may reflect a reduction in local membrane MerTK substrate rather than increased MerTK shedding on a per-cell basis. One of the major cellular sources of MerTK is the macrophage, which supports the resolution of inflammation; in periodontitis, impaired efferocytosis has been reported, characterized by a shift away from MerTK-rich pro-resolving phenotypes and the accumulation of uncleared apoptotic cells [5,27]. In this context, a reduced or reprogrammed pool of MerTK-expressing macrophages may release less ectodomain into GCF, even if proteolytic shedding is maintained. By contrast, macrophage-rich atherosclerotic plaques contain abundant membrane substrate, which may explain increased soluble Mer levels despite reduced surface MerTK expression [11]. In addition, unlike a closed lesion environment, GCF is a continuously formed, serum-derived fluid that is exposed to flow and washout. Therefore, low GCF sMerTK levels in periodontitis may indicate reduced local pro-resolving/efferocytic capacity rather than accelerated shedding. Conversely, higher sMerTK levels in periodontal health may be associated with preserved resolution capacity. This interpretation is also consistent with the reported pattern of developmental endothelial locus-1, a pro-resolving GCF component that is suppressed during active periodontal inflammation and increases after clinical resolution [29].

The interpretation of the Gas6 findings requires consideration of both its potential source and the metric used for analysis. TAM receptors and ligands have been shown to be locally expressed in healthy human masticatory mucosa, suggesting that periodontal tissues may represent a potential source of Gas6 [26]. However, the available tissue and cellular data do not support a marked increase in local Gas6 expression during periodontal inflammation. In gingival tissue and periodontal ligament cells, Gas6 expression has been reported to decrease or remain unchanged under inflammatory stimulation, while Porphyromonas gingivalis lipopolysaccharide can suppress Gas6 expression through NF-κB signaling [24,30]. Nevertheless, a previous study reported higher salivary GAS6 levels in periodontitis, and the present study also found an increased total amount of GCF Gas6 in the periodontitis group. This finding may be related to an increased serum-derived contribution rather than local overproduction. Indeed, Gas6 has been reported to increase in plasma in association with systemic inflammation, and some studies have suggested that it may exhibit behavior related to the acute-phase response [31]. In inflamed periodontal sites, increased GCF volume and vascular permeability may facilitate the entry of serum-derived ligands into GCF. Our data are consistent with this interpretation: the total amount of Gas6 increased only modestly, whereas Gas6 concentration decreased, a divergence that appears compatible with the expected pattern of a serum-derived soluble molecule entering a larger exudate volume. Moreover, Gas6 showed only weak-to-moderate correlations with clinical parameters, whereas sMerTK and the Gas6/sMerTK ratio showed stronger associations with GCF volume, gingival index, plaque index, and clinical attachment loss. Although a contribution from a local resolution response cannot be excluded, the available expression data do not support a marked local increase in Gas6 during disease. This source ambiguity has a bounded effect on interpretation. Because the serum-derived and locally produced fractions of GCF Gas6 cannot be separated by ELISA, the rise in Gas6 total amount should be read cautiously rather than as direct evidence of an enhanced local pro-resolving response. It does not, however, affect the central finding: the decline in sMerTK—a locally shed macrophage-derived fragment, not a molecule expected to enter GCF mainly through serum exudation—and the resulting increase in the Gas6/sMerTK ratio are not attributable to a serum-derived contribution, consistent with the weak correlations of Gas6, versus the strong correlations of sMerTK and the ratio, with clinical parameters.

Irrespective of its source, the increased Gas6/sMerTK ratio indicates a TAM axis in which ligand availability is preserved or modestly increased, whereas the soluble receptor marker is markedly reduced. Gas6 is a pro-resolving ligand that can induce SOCS1/3 expression through Axl and MERTK, thereby limiting NF-κB and STAT1 signaling and supporting efferocytosis and the resolution of inflammation [24,25]. Therefore, the marked decrease in sMerTK levels despite preserved or slightly increased Gas6 levels suggests that the impaired resolution response in periodontal lesions cannot be explained solely by ligand deficiency. In other inflammatory disease compartments, soluble TAM receptors have been reported to increase with disease and may act as decoy receptors by binding Gas6 and limiting its activity; this has been shown for soluble Mer in systemic lupus erythematosus [12,32] and atherosclerosis [11], and for soluble Axl in osteoarthritic synovial fluid and fibroblasts [25] as well as in multiple sclerosis lesions [33]. In contrast, sMerTK levels decreased in the present GCF pattern. Thus, low sMerTK is more likely to reflect insufficiency of the local MerTK-related receptor/effector arm, rather than increased Gas6 trapping. This interpretation is consistent with the reduced gingival Tyro3 expression reported in periodontitis [28] and the impaired macrophage efferocytosis described in disease [5,27]. Accordingly, the Gas6/sMerTK ratio may reflect a widening mismatch between available ligand and functional receptor response accompanying failed resolution of inflammation. However, because this interpretation is based on soluble surrogate markers, it requires validation at the tissue level through assessment of membrane MerTK expression and macrophage phenotypes.

Whether the altered Gas6/sMerTK ratio contributes to disease activity or merely reflects it cannot be resolved here. The ratio is a derived composite index rather than an effector molecule, so any biological action would reside in its components—chiefly the availability of functional, membrane-bound MerTK—rather than in the ratio itself. Moreover, the cross-sectional design cannot establish temporality: the decline in sMerTK may follow a disease-associated contraction or reprogramming of the MerTK-expressing macrophage pool [5,27], and the modest, apparently serum-derived rise in Gas6 may reflect inflammation-driven exudation rather than a local driving signal [31]. A contributory role nonetheless remains plausible, if the low soluble signal indexes a genuine deficit in the membrane MerTK–efferocytosis arm that helps sustain non-resolving inflammation. On current evidence, the ratio is therefore best regarded as an informative, exploratory marker of the periodontal resolution state rather than a demonstrated driver of disease.

From a clinical perspective, the stepwise separation of the three periodontal conditions by the Gas6/sMerTK ratio, together with the strong inverse associations between sMerTK and GCF volume, gingival index, plaque index, and clinical attachment loss, suggests that the local TAM balance may be closely related to the inflammatory and resolution status of the periodontium. Compared with the individual analytes, the Gas6/sMerTK ratio showed a more pronounced gradient across periodontal health, gingivitis, and periodontitis, and may therefore represent a potential composite GCF indicator of periodontal status. However, these findings should not be interpreted as a diagnostic claim. The study was exploratory and cross-sectional in design; threshold values and discriminatory performance were not assessed. Therefore, any potential biomarker application requires prospective validation before clinical use can be recommended.

The main limitation of this study is its cross-sectional design, which precludes determining whether reduced GCF sMerTK levels precede or follow periodontal destruction. The groups were not matched for age or sex; therefore, residual confounding cannot be excluded, particularly for analytes with a possible serum-derived component. In addition, ELISA measures total soluble protein levels and does not assess membrane MerTK expression, receptor function, or local efferocytosis. Single-center, site-based sampling at one time point and the absence of tissue, microbiological, and macrophage phenotype data should also be considered. Thus, the Gas6/sMerTK ratio should be interpreted as an exploratory indicator requiring prospective and mechanistic validation.

Future studies should assess GCF sMerTK levels before and after non-surgical periodontal therapy to determine whether this marker changes with the resolution of inflammation [29]. Integrating GCF findings with gingival tissue analyses of membrane MerTK expression, ADAM17 activity, and macrophage phenotypes may further clarify the mechanism underlying reduced sMerTK and whether increased Gas6 reflects local production or serum exudation.

## 5. Conclusions

In conclusion, this study suggests that the local Gas6/sMerTK balance in GCF is altered across periodontal health, gingivitis, and periodontitis. The progressive increase in the Gas6/sMerTK ratio was mainly driven by a marked reduction in sMerTK, indicating a possible disruption of the local TAM-related resolution axis in periodontal inflammation. Although the ratio may represent a promising exploratory indicator of periodontal status, these findings should be interpreted cautiously and require confirmation in longitudinal and mechanistic studies.

## Figures and Tables

**Table 1:** Comparison of demographic characteristics among the study groups.

	Periodontitis	Gingivitis	Periodontal Health	Total	Test Statistic	*p*	ES [%95 CI]
Sex							
Female	7 (25.9)	15 (55.6)	15 (55.6)	37 (45.7)	6.380	0.048 ^x^	0.280 [0.058 = 0.447]
Male	20 (74.1)	12 (44.4)	12 (44.4)	44 (54.3)			
Age (years)	46.3 ± 9.75 ^a^	32.59 ± 8.2 ^b^	35.63 ± 11.78 ^b^	38.17 ± 11.52	13.942	<0.001 ^y^	0.263 [0.101 = 0.398]

^x^ Monte Carlo Simulation Fisher’s Exact Test; ^y^ One-Way Analysis of Variance; ^a,b^ There is no difference between groups with the same letter; Mean ± Standard Deviation; frequency (percent); ES [%95 CI]: Effect size (%95 confidence interval): eta-squared (η^2^).

**Table 2:** Comparison of clinical periodontal parameters among the study groups.

	Periodontitis	Gingivitis	Periodontal Health	Test Statistic	*p*	ES [%95 CI]
GCF (μl)	0.75 (0.48: 1.13)/ 66.98 ^a^	0.39 (0.19: 0.75)/ 40.59 ^b^	0.17 (0.07: 0.48)/ 15.43 ^c^	64.878	<0.001 ^x^	0.806 [0.730: 0.870]
PD (mm)	3.32 ± 0.58 ^a^	1.67 ± 0.27 ^b^	1.65 ± 0.31 ^b^	96.703	<0.001 ^y^	0.789 [0.699: 0.837]
CAL (mm)	4.24 (2.69: 5.77)/68 ^a^	0 (0: 0)/27.5 ^b^	0 (0: 0)/27.5 ^b^	75.795	<0.001 ^x^	0.946 [0.910: 0.970]
PI	1.47 ± 0.24 ^a^	1.31 ± 0.18 ^b^	0.27 ± 0.1 ^c^	542.544	<0.001 ^y^	0.901 [0.856: 0.923]
GI	1.71 ± 0.19 ^a^	1.47 ± 0.12 ^b^	0.31 ± 0.1 ^c^	1043.113	<0.001 ^y^	0.951 [0.929: 0.962]

^x^ Kruskal–Wallis H Test; ^y^ One-Way Analysis of Variance; ^a–c^ No difference between groups with the same letter; Multiple comparisons were examined with Dunn and Games–Howell tests with Bonferroni correction; Mean ± standard deviation and median (minimum–maximum)/mean rank values are presented in this table; ES [%95 CI]: Effect size (%95 confidence interval): eta-squared (η^2^). CAL, clinical attachment loss; GCF, gingival crevicular fluid; GI, gingival index; PD, probing depth; PI, plaque index.

**Table 3:** Comparison of GCF Gas6, sMerTK, and Gas6/sMerTK ratio among the study groups.

	Periodontitis	Gingivitis	Periodontal Health	Test Statistic	*p*	ES [%95 CI]
Total Amounts						
Gas6 (ng)	0.22 (0.14: 0.3)/ 53.26 ^a^	0.2 (0.07: 0.24)/ 44.46 ^a^	0.16 (0.01: 0.23)/ 25.28 ^b^	19.975	<0.001 ^x^	0.230 [0.080: 0.440]
sMerTK (pg)	2.59 (0.04: 10.85)/ 16.91 ^a^	10.33 (0.49: 14.73)/ 39.93 ^b^	14.81 (10.55: 19.78)/ 66.17 ^c^	59.275	<0.001 ^x^	0.734 [0.630: 0.830]
Concentrations						
sMerTK (pg/μL)	3.57 (0.05: 17.22)/ 15.15 ^a^	23.08 (1.12: 55.54)/ 40.3 ^b^	90.68 (30.85: 216.06)/ 67.56 ^c^	67.025	<0.001 ^x^	0.834 [0.750: 0.880]
Gas6 (ng/μL)	0.29 (0.15: 0.42)/ 18.67 ^a^	0.5 (0.24: 0.89)/ 48.48 ^b^	0.77 (0.15: 2.61)/ 55.85 ^b^	37.821	<0.001 ^x^	0.459 [0.290: 0.640]
Gas6/sMerTK ratio (pg)	62.32 (13.71: 3470.38)/ 65.15 ^a^	20.9 (6.88: 409.41)/ 41.07 ^b^	9.86 (1.28: 19.91)/ 16.78 ^c^	57.066	<0.001 ^x^	0.706 [0.570: 0.800]

^x^ Kruskal–Wallis H Test; ^a–c^ Groups sharing the same letter are not significantly different; multiple comparisons were performed using Dunn’s test with Bonferroni correction. Median (minimum–maximum)/mean rank values are presented in this table; ES [%95 CI]: Effect size (%95 confidence interval): eta-squared (η^2^).

**Table 4:** Correlations between biomarker total amounts and clinical periodontal parameters.

	Gas6/sMerTK Ratio (pg)		sMerTK (pg) Total Amount		Gas6 (ng) Total Amount	
	r	*p*	r	*p*	r	*p*
sMerTK (pg) total amount	−0.933 ^x^	<0.001 ^x^	—	—	—	—
Gas6 (ng) total amount	0.641 ^x^	<0.001 ^x^	−0.453 ^x^	<0.001 ^x^	—	—
GCF (μl)	0.797 ^x^	<0.001 ^x^	−0.789 ^x^	<0.001 ^x^	0.506 ^x^	<0.001 ^x^
PD (mm)	0.582 ^x^	<0.001 ^x^	−0.609 ^x^	<0.001 ^x^	0.255 ^x^	0.022 ^x^
CAL (mm)	0.689 ^x^	<0.001 ^x^	−0.690 ^x^	<0.001 ^x^	0.340 ^x^	0.002 ^x^
PI	0.683 ^x^	<0.001 ^x^	−0.700 ^x^	<0.001 ^x^	0.373 ^x^	<0.001 ^x^
GI	0.775 ^x^	<0.001 ^x^	−0.771 ^x^	<0.001 ^x^	0.451 ^x^	<0.001 ^x^

^x^ Spearman correlation; CAL, clinical attachment loss; GCF, gingival crevicular fluid; GI, gingival index; PD, probing depth; PI, plaque index.

## Data Availability

The data supporting the findings of this study are not publicly available due to privacy and ethical restrictions, but may be made available from the corresponding author upon reasonable request and with permission from the relevant institutional authorities.

## Abbreviations

The following abbreviations are used in this manuscript:

Gas6	growth arrest-specific-6
GCF	gingival crevicular fluid
sMerTK	soluble MerTK
STROBE	Strengthening the Reporting of Observational Studies in Epidemiology
PD	probing depth
BOP	bleeding on probing
CAL	clinical attachment loss
RBL	radiographic bone loss
GI	gingival index
PI	plaque index
ELISA	enzyme-linked immunosorbent assay
ANOVA	one-way analysis of variance
